# Predictive Value of Triglyceride Glucose Index for the Risk of Incident Diabetes: A 4-Year Retrospective Longitudinal Study

**DOI:** 10.1371/journal.pone.0163465

**Published:** 2016-09-28

**Authors:** Da Young Lee, Eun Seo Lee, Ji Hyun Kim, Se Eun Park, Cheol-Young Park, Ki-Won Oh, Sung-Woo Park, Eun-Jung Rhee, Won-Young Lee

**Affiliations:** Division of Endocrinology and Metabolism, Department of Internal Medicine, Kangbuk Samsung Hospital, Sungkyunkwan University School of Medicine, Seoul, Korea; Mexican Social Security Institute, MEXICO

## Abstract

The Triglyceride Glucose Index (TyG index) is considered a surrogate marker of insulin resistance. The aim of this study is to investigate whether the TyG index has a predictive role in identifying individuals with a high risk of incident diabetes and to compare it with other indicators of metabolic health. A total 2900 non-diabetic adults who attended five consecutive annual health check-ups at Kangbuk Samsung Hospital was divided into four subgroups using three methods: (1) baseline TyG index; (2) obesity status (body mass index ≥25 kg/m^2^) and cutoff value of TyG index; (3) obesity status and metabolic health, defined as having fewer than two of the five components of high blood pressure, fasting blood glucose, triglyceride, low high-density lipoprotein cholesterol, and highest decile of homeostasis model assessment-insulin resistance. The development of diabetes was assessed annually using self-questionnaire, fasting glucose, and glycated hemoglobin. We compared the risk of incident diabetes using multivariate Cox analysis. During 11623 person-years there were 101 case of incident diabetes. Subjects with high TyG index had a high risk of diabetes. For TyG index quartiles, hazard ratios (HRs) of quartiles 3 and 4 were 4.06 (*p* = 0.033) and 5.65 (*p* = 0.006) respectively. When the subjects were divided by obesity status and cutoff value of TyG index of 8.8, the subgroups with TyG index ≥ 8.8 regardless of obesity had a significantly high risk for diabetes (HR 2.40 [*p* = 0.024] and 2.25 [*p =* 0.048]). For obesity status and metabolic health, the two metabolically unhealthy subgroups regardless of obesity had a significantly high risk for diabetes (HRs 2.54 [*p* = 0.024] and 2.73 [*p* = 0.021]). In conclusion, the TyG index measured at a single time point may be an indicator of the risk for incident diabetes. The predictive value of the TyG index was comparable to that of metabolic health.

## Introduction

Obesity is recognized as an independent risk factor for cardiovascular disease and type 2 diabetes mellitus[1–3]. However, unexpected relationships between obesity and metabolic abnormalities have been reported. The metabolic disturbances generally associated with obesity may not be present in all obese individuals, and some non-obese individuals present abnormal metabolic findings that are typically related to obesity[4–6]. These subpopulations are called “metabolically healthy obese” and “metabolically unhealthy non-obese” individuals, respectively[7, 8].

A few researchers have noted that metabolically unhealthy non-obese individuals have an increased risk for type 2 diabetes, cardiovascular disease, and mortality[4, 7–14]. In one study, a metabolically unhealthy status was as an independent risk factor for diabetes regardless of obesity status[4]. In that study, metabolic health was defined by blood pressure (BP), lipid profile, fasting glucose, and waist circumference (WC).

Recently, a simple assessment for metabolic abnormality, the triglyceride glucose Index (TyG index), has been suggested in several studies[15–17]. This index is the product of the fasting blood glucose and triglyceride levels, which correlates with the degree of insulin resistance[16, 18].

In this retrospective longitudinal study, we aimed to investigate whether the TyG index had a predictive role in identifying individuals with a high risk of incident diabetes and to compare it with the predictive role of metabolic health.

## Materials and Methods

### Subjects

We investigated the medical records of adults aged 20 years or older who participated in medical health checkup programs at the Health Promotion Center of Kangbuk Samsung Hospital, Sungkyunkwan University, Seoul, Korea. Most of the examinees were employees and family members of various industrial companies from all over the country. The purpose of the medical health checkup program is to promote the health of employees through regular health checkups and to enhance early detection of existing diseases. These medical examinations are largely paid for by the employers and a considerable proportion of the examinees undergo examinations annually or biannually.

Initially, 10868 subjects who attended five consecutive annual health checkups between January 2005 and December 2009 were assessed for eligibility. Among these, 7968 subjects were excluded because of the presence of diabetes or missing data, especially fasting insulin levels, lipid profiles, and WC. Final analyses were performed in 2,900 subjects (2078 men and 822 women) with mean age of 44.3 ± 6.5 years.

The subjects provided their written informed consent for use of their health screening data in the research. This study was reviewed and approved by the Institutional Review Board of Kangbuk Samsung Hospital (KBS12089) and was carried out in accordance with the Helsinki Declaration of 1975.

### Anthropometric and laboratory measurements

Each subject completed a structured questionnaire addressing demographic characteristics at the first visit. Height and weight were each measured twice and averaged. The body mass index (BMI) of subjects was calculated as weight in kilograms divided by the square of height in meters. Waist circumference (WC) was measured in the standing position at the middle point between the anterior iliac crest and the lower border of the rib by a single examiner.

Body composition measurements of the subjects were carried out by segmental bioelectric impedance using eight tractile electrodes according to the manufacturer’s instructions (InBody 3·0, Biospace, Korea). Lean mass (kg), fat mass (kg), and percent fat mass (%) were measured. Skeletal muscle index (SMI) was calculated with the following formula: lean mass (kg) / body weight (kg) × 100[19]. Blood pressure (BP) was measured using a standardized sphygmomanometer after 5 minutes of rest. Systolic BP and diastolic BP were measured three times with participants in the seated position, with 1 minute of rest between each measurement. The average of the second and third measurements was used in the analysis.

Venous blood samples were collected in the morning (8–9 am) after an overnight fast of more than 8 hours. The hexokinase method was used to test fasting glucose concentrations (Hitachi Modular D2400; Roche, Tokyo, Japan). Fasting insulin concentrations were determined by electrochemiluminescence immunoassay (Hitachi Modular E170; Roche). Aspartate aminotransferase (AST) and alanine aminotransferase (ALT) were measured by UV without the P5P method (Advia 1650 Autoanalyzer, Bayer diagnostics, Leverkusen, Germany). An enzymatic calorimetric test was used to measure the total cholesterol (TC) and triglyceride (TG) concentrations. The selective inhibition method was used to measure the level of high-density lipoprotein cholesterol (HDL-C), and a homogeneous enzymatic calorimetric test was used to measure the level of low-density lipoprotein cholesterol (LDL-C). Glycosylated hemoglobin (HbA1c) was measured by immunoturbidimetric assay with a Cobra Integra 800 automatic analyzer (Roche Diagnostics, Basel, Switzerland) with a reference value of 4.4–6.4%. The methodology was aligned with the Diabetes Control and Complications Trial and National Glycohemoglobin Standardization Program standards[20]. The intra-assay coefficient of variation (CV) was 2.3% and inter-assay CV was 2.4%; both were within the NGSP acceptable limits[21]. Serum high-sensitivity C-reactive protein (hsCRP) concentrations were measured using a nephelometric assay and a BNII nephelometer (Dade Behring, Deerfield, IL, USA). Insulin resistance was measured using the homeostatic model of the assessment of insulin resistance (HOMA-IR) and was obtained by applying the following formula: HOMA-IR = fasting insulin (IU/mL) × fasting blood glucose (mmol/L)/22.5[22].

Smoker status was defined as a subject who had ever smoked at least five total packs of cigarettes in his/her whole life. Alcohol drinking was defined as drinking more than 20 g of alcohol every day. Regular exercise was defined as regular exercise of moderate intensity at least three times a week. These lifestyle habits were assessed annually by a self-questionnaire.

Hypertension was defined as systolic blood pressure (SBP) ≥140 mmHg, diastolic blood pressure (DBP) ≥90 mmHg, or the current use of antihypertensive medications according to criteria recommended by the seventh report of the Joint National Committee on prevention, detection, evaluation, and treatment of high BP (JNC 7)[23].

Based on the criteria of the American Diabetes Association (ADA)[24], diabetes was defined as levels of fasting glucose ≥ 126 mg/dL or HbA1c ≥ 6.5% and/or the current use of antihyperglycemic medications. Development of diabetes was assessed at every yearly examination with the same diagnostic criteria for diabetes mellitus.

Determination of the TyG index was based on the formula: ln (fasting TG [mg/dL] × fasting glucose [mg/dL]/2)[16, 18].

### Definitions of obesity and metabolic health

In 2000, the WHO Western Pacific Region suggested revised Asia–Pacific criteria of obesity in Asian populations using reduced values of BMI in both sexes[25]. Individuals with BMI ≥ 25 kg/m^2^ were classified as obese, whereas others were classified as non-obese.

Metabolic healthy was defined as having fewer than two abnormalities among the standard components of metabolic risk factors using the modified criteria proposed by Wildman et al., which replaces WC with insulin resistance status defined by HOMA-IR [5, 26]. The five criteria used in this study were (1) SBP ≥ 130 mmHg and/or DBP ≥ 85 mmHg or on antihypertensive treatment; (2) TG ≥150 mg/dL; (3) Fasting glucose ≥ 100 mg/dL; (4) HDL-C < 40 mg/dL in men, < 50 mg/dL in women; and (5) HOMA-IR ≥ 90th percentile.

### Statistical analysis

Baseline characteristics were calculated for the total number of subjects. All data were presented as mean ± SD or as proportions. Because serum AST, ALT, TG, hsCRP, insulin, and HOMA-IR levels were not normally distributed, AST, ALT, TG, and hsCRP were converted to logarithmic values (Ln) and HOMA-IR were converted to square root values for analysis.

The subjects were stratified into four groups based on baseline TyG index. Baseline characteristics of the quartiles were compared with a reference group by ANOVA for continuous variables and by chi-square tests for categorical variables. Multivariate Cox proportional hazards analysis was used to assess the relative risk of diabetes according to quartiles of TyG Index. To adjust for confounders, we used three models: model 1 was adjusted for age and sex; model 2 was additionally adjusted for baseline history of smoking, alcohol drinking, and regular exercise status; and model 3 was additionally adjusted for SBP, HDL-C, LDL-C, HOMA-IR, and hsCRP. Kaplan-Meier survival analyses were performed for incident diabetes development after 4 years according to the TyG index quartiles.

The validity of the proportional hazards assumption was evaluated by inspection of Schoenfeld residuals versus time. No associations between residuals and time were present therefore we could not reject the proportionality assumption. To avoid multicollinearity, we assessed the variable inflation factor (VIF) for all covariates included in each of the regression models. All variables had a VIF less than 2.0, indicating no relevant multicollinearity among covariates[27]. *P* values were corrected by Bonferroni’s method because of multiple testing.

We also repeated the above analysis after dividing the total subjects according to the cutoff value of TyG Index and obesity status as follows: (1) BMI < 25 kg/m^2^ and TyG Index < cutoff value; (2) BMI < 25 kg/m^2^ and TyG Index ≥ cutoff value; (3) BMI ≥ 25 kg/m^2^ and TyG Index < cutoff value; (4) BMI ≥ 25 kg/m^2^ and TyG Index ≥ cutoff value.

For comparison, we additionally divided the subjects according to obesity status and metabolic health as in a previous study[4]: (1) metabolically healthy, non-obese (MHNO): BMI < 25 kg/m^2^ and < 2 metabolic risk factors; (2) metabolically unhealthy, non-obese (MUHNO): BMI < 25 kg/m^2^ and ≥ 2 metabolic risk factors; (3) metabolically healthy, obese (MHO): BMI ≥ 25 kg/m^2^ and < 2 metabolic risk factors; (4) metabolically unhealthy, obese (MUHO): BMI ≥ 25 kg/m^2^ and ≥ 2 metabolic risk factors.

The areas under the receiver operating characteristics (ROC) curves and 95% confidence interval (CI) were calculated to define the cutoff value of the TyG index at the final visit that predicted diabetes. The optimal cutoff value was determined from the maximal Youden’s Index (sensitivity + specificity—1).

Statistical software SPSS version 21.0 software (Chicago, IL, USA) was used for statistical analysis. A value of *p* < 0.05 was considered statistically significant.

## Results

Baseline characteristics of total subjects are described in S1 Table. The mean age of total subjects was 44.3 ± 6.5. Among these, 944 subjects (32.6%) were obese (BMI ≥ 25 kg/m^2^), 1,194 (41.2%) were metabolically unhealthy, and 836 (28.8%) had impaired fasting glucose.

Subjects were stratified into TyG index quartiles as shown in Table 1. As TyG index quartiles increased, metabolic parameters tended to get worse: BMI, WC, TC, TG, LDL-C, fasting insulin, and HOMA-IR increased, while HDL-C decreased. In addition, the proportion of males and the prevalence of smoking, alcohol drinking, and metabolically unhealthy status increased.

**Table 1:** Baseline characteristics and their comparisons according to TyG index quartiles.

Variables	TyG Index Quartiles				*P* value*
	Quartile 1 (< 8.21) n = 725	Quartile 2 (8.21~8.56) n = 721	Quartile 3 (8.57~8.96) n = 729	Quartile 4 (≥ 8.97) n = 725	
Age (years)	43.3 ± 6.7	44.8 ± 6.6^#^	45.1 ± 7.0^#^	44.1 ± 5.5	< 0.001
Sex, male (%)	316 (43.6)	511 (70.9) ^#^	589 (80.8) ^#^	662 (91.3) ^#^	< 0.001
BMI (kg/m^2^)	21.9 ± 2.4	23.4 ± 2.6^#^	24.5 ± 2.6^#^	25.3 ± 2.6^#^	< 0.001
Waist circumference (cm)	74.3 ± 8.1	79.8 ± 8.4^#^	83.2 ± 8.0^#^	86.1 ± 7.0^#^	< 0.001
Lean mass (kg)	43.1 ± 8.0	47.7 ± 8.2^#^	49.8 ± 7.8^#^	52.3 ± 7.1^#^	< 0.001
Body fat mass (kg)	14.4 ± 4.2	15.7 ± 4.4^#^	17.3 ± 4.5^#^	18.1 ± 4.4^#^	< 0.001
Percent body fat (%)	24.0 ± 6.1	23.7 ± 5.9	24.7 ± 5.4	24.5 ± 4.5	0.003
Systolic BP (mmHg)	107.3 ± 12.6	112.3 ± 14.9^#^	114.9 ± 14.4^#^	116.2 ± 14.8^#^	< 0.001
Diastolic BP (mmHg)	71.6 ± 9.2	76.4 ± 10.3^#^	77.9 ± 9.8^#^	80.1 ± 10.0^#^	< 0.001
Total cholesterol (mg/dL)	180.4 ± 29.4	191.0 ± 29.0^#^	197.4 ± 32.7^#^	209.1 ± 35.1^#^	< 0.001
Triglyceride (mg/dL)	63.7 ± 12.1	94.0 ± 11.6^#^	134.2 ± 18.5^#^	240.3 ± 99.0^#^	< 0.001
HDL-C (mg/dL)	59.5 ± 13.0	55.2 ± 11.4^#^	50.4 ± 10.0^#^	46.9 ± 9.1^#^	< 0.001
LDL-C (mg/dL)	102.0 ± 24.2	112.3 ± 24.8^#^	118.0 ± 28.6^#^	116.4 ± 29.8^#^	< 0.001
Hemoglobin A1c (%)	5.4 ± 0.3	5.4 ± 0.3	5.4 ± 0.3^#^	5.5 ± 0.3^#^	< 0.001
Fasting glucose (mg/dl)	91.4 ± 7.1	95.0 ± 8.0^#^	96.8 ± 8.7^#^	99.6 ± 9.0^#^	< 0.001
Fasting insulin (IU/L)	7.6 ± 2.6	8.2 ± 2.9^#^	9.1 ± 3.2^#^	10.3 ± 4.0^#^	< 0.001
HOMA-IR	1.73 ± 0.64	1.93 ± 0.73^#^	2.20 ± 0.81^#^	2.54 ± 1.09^#^	< 0.001
hsCRP (mg/mL)	0.09 ± 0.29	0.15 ± 0.65^#^	0.13 ± 0.34^#^	0.12 ± 0.19^#^	< 0.001
Smoking (%)^a^	222 (31.3)	319 (45.1) ^#^	440 (61.5) ^#^	536 (75.3) ^#^	< 0.001
Alcohol drinking (%)	38 (5.2)	76 (10.5) ^#^	81 (11.1) ^#^	112 (15.4) ^#^	< 0.001
Regular exercise (%)	174 (24.0)	203 (28.2)	153 (21.0)	117 (16.1) ^#^	< 0.001
IFG (%)	94 (13.0)	176 (24.4)^#^	242 (33.2)^#^	324 (44.7)^#^	< 0.001
Metabolically unhealthy status (%)	64 (8.8)	164 (22.7)^#^	320 (43.9)^#^	646 (89.1)^#^	< 0.001
TyG index	7.95 ± 0.21	8.39 ± 0.10^#^	8.77 ± 0.11^#^	9.33 ± 0.32^#^	< 0.001

Data are presented as frequency (%), mean ± SD.

TyG, the products of triglycerides and fasting glucose; BMI, body mass index; BP, blood pressure; AST, aspartate aminotransferase; ALT, alanine aminotransferase; BUN, blood urea nitrogen; HDL-C, high-density lipoprotein cholesterol; LDL-C, low-density lipoprotein cholesterol; HbA1c, glycosylated hemoglobin; HOMA-IR, homeostasis model assessment index-insulin resistance; hsCRP, high-sensitivity C-reactive protein; IFG, impaired fasting glucose

^a^ Subjects who have ever smoked more than 5 packs of cigarettes.

Smoking history was available only in 2845 subjects, divided into groups of 710, 707, 716, and 712 subjects.

* *P* values were derived from one-way ANOVA analysis and chi-square tests.

^#^ *P* < 0.05, in comparison with the reference group (quintile 1). *P* values were corrected by Bonferroni’s method due to multiple testing.

AST, ALT, triglyceride, fasting insulin, and hsCRP were converted to Ln values and HOMA-IR was converted to square root value for the analysis.

During 11623 person-years of follow-up (median follow-up 48.5 months), there were 101 cases of incident diabetes. Table 2 shows hazard ratio (HR) and 95% CI values for the development of diabetes according to TyG index quartiles. The proportions of subjects with incident diabetes during the follow-up period increased across TyG index quartiles, and quartile 4 had a significantly higher risk of diabetes compared with quartile 1 (reference group). These associations persisted in all adjusted models (models 1–3) with the exception of quartile 2, which no longer showed significance in the multivariate model. In fully adjusted model 3, the HRs for diabetes of quartiles 3 and 4 compared to quartile 1 were 4.06 (95% CI 1.39–11.88, *p* value = 0.033) and 5.65 (95% CI 1.91–16.73, *p* value = 0.006), respectively.

**Table 2:** Hazard ratios of incident diabetes according to TyG Index quartiles.

	*n*	Diabetes, *n* (%)	Univariate HR (95% CI)	Multivariate HR (95% CI)		
				Model 1	Model 2	Model 3
< 8.21	725	4 (0.6)	1.00 (ref.)	1.00 (ref.)	1.00 (ref.)	1.00 (ref.)
*p* value*			-	-	-	-
8.21~8.56	721	16 (2.2)	4.07 (1.36–12.16)	3.39 (1.13–10.20)	3.17 (1.04–9.61)	2.61 (0.86–7.96)
*p* value*			0.036	0.090	0.126	0.091
8.57~8.96	729	31 (4.3)	7.70 (2.72–21.80)	5.87 (2.05–16.81)	5.86 (2.04–16.82)	4.06 (1.39–11.88)
*p* value*			< 0.001	0.003	0.003	0.033
≥ 8.97	725	50 (6.9)	12.62 (4.56–34.93)	10.38 (3.68–29.28)	10.26 (3.63–29.07)	5.65 (1.91–16.73)
*p* value*			< 0.001	< 0.001	< 0.001	0.006

Model 1 adjusted for age and sex; model 2 adjusted for model 1 plus baseline history of smoking, alcohol drinking, and regular exercise status; model 3 adjusted for model 2 plus systolic blood pressure, HDL-C, LDL-C, HOMA-IR, and hsCRP.* *P* values were corrected by Bonferroni’s method due to multiple testing.HOMA-IR was converted to square root value and hsCRP was converted to Ln values for the analysis.TyG, the products of triglycerides and fasting glucose; HR, hazard ratio; CI, confidence interval; HDL-C, high-density lipoprotein cholesterol; LDL-C, low-density lipoprotein cholesterol; HOMA-IR, homeostasis model assessment index—insulin resistance; hsCRP, high-sensitivity C-reactive protein

**Fig 1:**
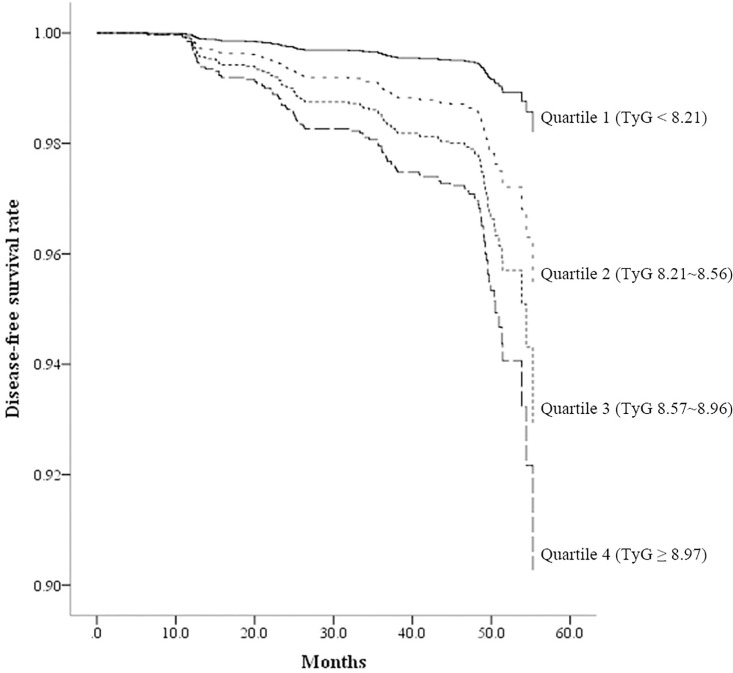
Disease-free survival by Kaplan-Meier analysis. Median follow-up period was 48.5 months. Subjects were divided into four groups according to baseline TyG Index.

In Kaplan-Meier disease-free survival analysis, among the four groups quartile 4 showed the lowest disease-free survival for diabetes and quartile 1 showed the highest disease-free survival (Fig 1).

To determine the cutoff value of the TyG index for identifying the development of diabetes, a receiver operating characteristics analysis was performed (S1 Fig). The optimal cutoff value was 8.8, with the areas under the ROC curves value of 0.751 (95% CI 0.704–0.799).

Table 3 shows baseline characteristics and their comparisons according to obesity status and cutoff value of TyG index 8.8. Considering BMI, WC, SBP, DBP, lipid profiles, HOMA-IR, the portions of males, smoking, alcohol drinking, and metabolic health, the group with BMI ≥ 25 kg/m^2^ and TyG Index ≥ 8.8 had the worst metabolic profiles, whereas the group with BMI < 25 kg/m^2^ and TyG Index < 8.8 was the most metabolically healthy group. Moreover, groups with TyG < 8.8 irrespective of BMI were more metabolically healthy than those with TyG ≥ 8.8.

**Table 3:** Baseline characteristics and their comparisons according to obesity status and TyG index.

Variables	BMI < 25 cm/m^2^		BMI ≥ 25 cm/m^2^		P value^*^
	TyG < 8.8 (n = 1445)	TyG ≥ 8.8 (n = 511)	TyG < 8.8 (n = 424)	TyG ≥ 8.8 (n = 520)	
Age (years)	44.1 ± 6.8	44.6 ± 6.4	44.7 ± 6.5	44.2 ± 5.8	0.285
Sex, male (%)	816 (56.5)	423 (82.8)^§^	350 (82.5)^#^	489 (94.0)^#^^,^^§^	< 0.001
BMI (kg/m^2^)	21.9 ± 1.9	23.0 ± 1.5^§^	26.7 ± 1.5^#^	27.1 ± 1.8^#^^,^^§^	< 0.001
Waist circumference (cm)	75.5 ± 7.4	80.9 ± 6.1^§^	88.3 ± 6.0^#^	89.8 ± 5.6^#^^,^^§^	< 0.001
Lean mass (kg)	44.3 ± 7.8	48.3 ± 7.0^§^	53.0 ± 7.1^#^	55.1 ± 6.4^#^^,^^§^	< 0.001
Body fat mass (kg)	14.0 ± 3.4	15.0 ± 2.8^§^	20.6 ± 3.9^#^	20.7 ± 3.8^#^	< 0.001
Percent body fat (%)	23.2 ± 5.7	22.9 ± 4.7	27.1 ± 5.2^#^	26.3 ± 4.3^#^^,^^§^	< 0.001
Systolic BP (mmHg)	109.2 ± 13.7	113.0 ± 14.3^§^	116.1 ± 13.9^#^	119.2 ± 15.0^#^^,^^§^	< 0.001
Diastolic BP (mmHg)	73.3 ± 9.7	77.0 ± 9.3^§^	80.0 ± 9.6^#^	82.0 ± 10.1^#^^,^^§^	< 0.001
Total cholesterol (mg/dL)	186.5 ± 30.4	204.4 ± 33.9^§^	192.9 ± 29.3^#^	208.2 ± 36.3^#^^,^^§^	< 0.001
Triglyceride (mg/dL)	85.7 ± 25.6	207.5 ± 89.2^§^	100.3 ± 24.6^#^	218.4 ± 97.1^#^^,^^§^	< 0.001
HDL-C (mg/dL)	57.2 ± 12.4	48.2 ± 9.6^§^	51.5 ± 10.8^#^	47.2 ± 9.0^#^^,^^§^	< 0.001
LDL-C (mg/dL)	107.2 ± 25.5	114.7 ± 29.5^§^	117.1 ± 25.2^#^	119.6 ± 30.6^#^	< 0.001
HbA1c (%)	5.4 ± 0.3	5.4 ± 0.3^§^	5.4 ± 0.3	5.5 ± 0.3^#^^,^^§^	< 0.001
Fasting glucose (mg/dl)	93.3 ± 7.9	97.4 ± 8.9^§^	95.8 ± 8.0^#^	100.5 ± 8.9^#^^,^^§^	< 0.001
Fasting insulin (IU/L)	7.7 ± 2.6	8.8 ± 3.0^§^	9.6 ± 3.4^#^	11.2 ± 4.1^#^^,^^§^	< 0.001
HOMA-IR	1.79 ± 0.65	2.12 ± 0.6^§^	2.27 ± 0.86^#^	2.80 ± 1.13^#^^,^^§^	< 0.001
hsCRP (mg/mL)	0.11 ± 0.40	0.09 ± 0.18^§^	0.18 ± 0.64^#^	0.15 ± 0.33^#^	< 0.001
Smoking (%)^a^	559 (39.5)	328 (66.0)^§^	238 (56.5)^#^	392 (76.4)^#^^,^^§^	< 0.001
Alcohol drinking (%)	111 (7.7)	75 (14.7)^§^	41 (9.7)	80 (15.4)^#^^,^^§^	< 0.001
Regular exercise (%)	355 (24.6)	90 (17.6)^§^	112 (26.4)	90 (17.3)^#^^,^^§^	< 0.001
IFG (%)	280 (19.4)	190 (37.2)^§^	115 (27.1)^#^	251 (48.3)^#^^,^^§^	< 0.001
Metabolically unhealthy status (%)	207 (14.3)	388 (75.9)^§^	151 (35.6)^#^	448 (86.2)^#^^,^^§^	< 0.001
TyG index	8.24 ± 0.32	9.16 ± 0.32^#^^,^^§^	8.44 ± 0.27^#^	9.23 ± 0.35^#^^,^^§^	< 0.001

Data are presented as frequency (%), mean ± SD.

BMI, body mass index; TyG, the products of triglycerides and fasting glucose; BP, blood pressure; AST, aspartate aminotransferase; ALT, alanine aminotransferase; BUN, blood urea nitrogen; HDL-C, high-density lipoprotein cholesterol; LDL-C, low-density lipoprotein cholesterol; HbA1c, glycosylated hemoglobin; HOMA-IR, homeostasis model assessment index—insulin resistance; hsCRP, high-sensitivity C-reactive protein; IFG, impaired fasting glucose

^a^ Subjects who have ever smoked more than 5 packs of cigarettes.

Smoking history was available only in 2845 subjects, divided into groups of 1414, 497, 421, and 513 subjects.

* *P* values were derived from one-way ANOVA analysis and chi-square tests.

^§^ *p* < 0.05, in comparison with the group with TyG < 8.8 from one-way ANOVA analysis.

^#^ *p* < 0.05, in comparison with the reference group (BMI < 25 kg/m^2^ and TyG < 8.8) from one-way ANOVA analysis.

*P* values were corrected by Bonferroni’s method due to multiple testing.

AST, ALT, triglyceride, fasting insulin, and hsCRP were converted to Ln values and HOMA-IR was converted to square root value for the analysis.

**Fig 2:**
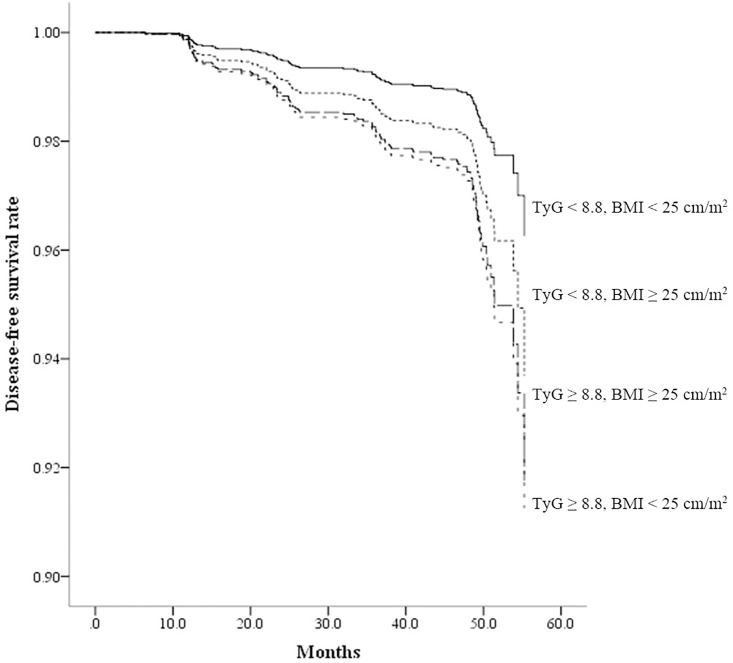
Disease-free survival by Kaplan-Meier analysis. Median follow-up period was 48.5 months. Subjects were divided into four groups according to obesity status and TyG index.

As shown in Table 4, the group with BMI < 25 kg/m^2^ and TyG index < 8.8 (the reference group) showed the lowest rate for incident diabetes, and the group with BMI ≥ 25 kg/m^2^ and TyG index ≥ 8.8 showed the highest rate for incident diabetes. Compared with the reference group, the other three groups showed a significantly increased risk for incident diabetes in univariate Cox proportional hazards analysis. However, in fully adjusted model 3, only the two groups with TyG index ≥ 8.8 regardless of obesity retained significance. The HRs of the subgroups with BMI < 25 kg/m^2^ and TyG index ≥ 8.8 and the subgroup with BMI ≥ 25 kg/m^2^ and TyG index ≥ 8.8 were 2.40 (95% CI 1.26–4.56, *p* value = 0.024) and 2.25 (95% CI 1.17–4.36, *p* value = 0.048), respectively. In Kaplan-Meier disease-free survival analysis, the group with low TyG index and non-obese status showed the highest disease-free survival among the four groups (Fig 2).

**Table 4:** Hazard ratios of incident diabetes according to obesity status and TyG Index or metabolic health.

	*n*	Diabetes, *n* (%)	Univariate HR (95% CI)		Multivariate HR (95% CI)		
					Model 1	Model 2	Model 3
Obesity status and TyG Index							
BMI < 25 kg/m^2^							
TyG < 8.8	1445	20 (1.4)	1.00 (ref.)		1.00 (ref.)	1.00 (ref.)	1.00 (ref.)
*p* value*			-		-	-	-
TyG ≥ 8.8	511	23 (4.5)	3.29 (1.81–5.99)		2.91 (1.59–5.34)	3.07 (1.65–5.72)	2.40 (1.26–4.56)
*p* value*			< 0.001		0.003	< 0.001	0.024
BMI ≥ 25 kg/m^2^							
TyG < 8.8	424	18 (4.2)	3.03 (1.60–5.72)		2.59 (1.36–4.94)	2.70 (1.40–5.20)	1.71 (0.87–3.37)
*p* value*			0.003		0.012	0.009	0.122
TyG ≥ 8.8	520	40 (7.7)	5.48 (3.20–9.37)		4.76 (2.73–8.32)	4.90 (2.76–8.69)	2.25 (1.17–4.36)
*p* value*			< 0.001		< 0.001	< 0.001	0.048
Obesity status and metabolic health							
MHNO	1361	16 (1.2)		1.00 (ref.)	1.00 (ref.)	1.00 (ref.)	1.00 (ref.)
*p* value*				-	-	-	-
MUHNO	595	27 (4.5)		3.80 (2.05–7.05)	3.23 (1.72–6.07)	3.36 (1.76–6.41)	2.54 (1.27–5.06)
*p* value*				< 0.001	< 0.001	< 0.001	0.024
MHO	345	10 (2.9)		2.42 (1.10–5.33)	2.17 (0.98–4.81)	2.27 (1.01–5.08)	1.64 (0.72–3.73)
*p* value*				0.084	0.057	0.141	0.235
MUHO	599	48 (8.0)		6.65 (3.78–11.72)	5.56 (3.08–10.05)	5.72 (3.12–10.50)	2.73 (1.31–5.68)
*p* value*				< 0.001	< 0.001	< 0.001	0.021

Model 1 adjusted for age and sex; model 2 adjusted for model 1 plus baseline history of smoking, alcohol drinking, and regular exercise status; model 3 adjusted for model 2 plus systolic blood pressure, HDL-C, LDL-C, HOMA-IR, and hsCRP.* *P* values were corrected by Bonferroni’s method due to multiple testing.hsCRP was converted to Ln values and HOMA-IR was converted to square root value for the analysis.TyG, the products of triglycerides and fasting glucose; HR, hazard ratio; CI, confidence interval; MHNO, metabolically healthy non-obese; MUHNO, metabolically unhealthy non-obese; MHO, metabolically healthy obese; MUHO, metabolically unhealthy obese; HDL-C, high-density lipoprotein cholesterol; LDL-C, low-density lipoprotein cholesterol; HOMA-IR, homeostasis model assessment index—insulin resistance; hsCRP, high-sensitivity C-reactive protein

When subjects were divided by obesity status and metabolic health, similar findings were observed. The MUHO group had the worst metabolic profiles and showed the highest rate for incident diabetes by 8.0%, whereas the MHNO group (the reference group) was the most metabolically healthy and showed the lowest rate for incident diabetes by 1.2%. In both univariate and multivariate analysis, the MUHNO group and MUHO group showed a significantly increased risk for incident diabetes among the four groups. The HRs of MUHNO and MUHO subgroups were 2.54 (95% CI 1.27–5.06, *p* value = 0.024) and 2.73 (95% CI 1.31–5.68, *p* value = 0.021), respectively (S2 Table and Table 4). In Kaplan-Meier disease-free survival analysis, the MUNO group showed the highest disease-free survival and the MUHO group showed the lowest disease-free survival among the four groups.

## Discussion

In this longitudinal study, we found that high baseline TyG index was related to the development of diabetes. This relationship was maintained irrespective of obesity status. Moreover, the predictive value of the TyG index was comparable to that of metabolic health.

The TyG index was proposed by Guerrero-Romero et al. as a surrogate marker of insulin resistance measured by the hyperinsulinemic–euglycemic clamp test[16, 18] This correlation was similar between men and women, non-obese and obese, and non-diabetic and diabetic individuals. Because hypertriglyceremia interferes with glucose metabolism in muscle, which is the major organ of insulin action and glucose uptake, the TyG index seems to mainly reflect muscle insulin resistance whereas HOMA-IR mainly reflects hepatic insulin resistance[28, 29].

Given that insulin resistance is the core pathophysiologic mechanism of type 2 diabetes, the TyG index has been proposed to aid the prediction of incident diabetes. Two prospective studies on the role of the TyG index as a predictive marker of future diabetes were conducted in one large-scale cohort[7, 17]. Subjects with high TyG index consistently had the highest incidence of diabetes (14.8%), followed by subjects whose TyG index was low and increased after 4 years (10.2%). Subjects whose TyG index decreased did not have an increased risk of diabetes. Compared with other indices of insulin resistance (TG/HDL-C ratio and HOMA-IR), the TyG index proved to be a better tool for predicting the development of diabetes with higher relative risk[17]. However, in this study cohort the rate of follow-up loss was relatively high (nearly 50%), serum HbA1c concentrations were not measured, and the interval of follow-up was 4 years. In addition, diagnosis of diabetes was based on only an oral glucose tolerance test.

In our study, subjects attended five consecutive annual health checkups. Diagnosis of diabetes was based on ADA criteria in both baseline and follow-up periods. As TyG index quartiles increased, obesity-related parameters were aggravated and the mean HOMA-IR and insulin levels increased. Subjects in TyG quartile 4 had the highest risk for diabetes during the 5-year follow-up. Even after adjusting for several known risk factors of diabetes and insulin level, the association between the baseline TyG index and the risk of future diabetes remained statistically significant, indicating that the TyG index is an independent risk factor. In multivariate analysis, the incidence rate was approximately 2-fold higher in subjects in quartiles 2 and 3 for TyG and more than 4-fold higher in subjects in quartile 4 in this study population. These findings are in accordance with previous reports and indicate that TyG index measured at a single time point may be an indicator of the risk for developing diabetes.

Deviation from the typical dose-response relationship between BMI representing simple obesity and metabolic disturbances has been observed in various ethnic groups[4–6]. Two unique subsets of obese individuals have been identified: MUHNO and MHO. MUHNO individuals display a cluster of obesity-related metabolic abnormalities but are not obese whereas MHO individuals seem to be protected against obesity-induced deterioration of metabolism. Thus, MUHNO and MHO seem to represent each end of the spectrum of obesity[30].

Previous studies have investigated the impact of BMI and metabolic health status[4–6]. Metabolic health was defined in accordance with established criteria for metabolic syndrome from various organizations[4–6, 9, 10, 15] that consisted of WC, TG, HDL-C, blood pressure, and fasting glucose. However, there is no consensus for the definite criteria for metabolic syndrome, and there has been considerable disagreement, especially concerning waist circumference. As a result, Wildman et al. modified the criteria by replacing WC with HOMA-IR[5, 26]. These modified criteria were used in the current study.

Many researchers have investigated simple definitions of metabolic abnormality. A recent study suggested the TyG index[15]. However, they defined “metabolically unhealthy” in three different ways: having metabolic syndrome; TyG index higher than the cutoff value (8.82 in males and 8.73 in females); or HOMA-IR in the highest quartile. Instead of this complicated definition, we divided our subjects into four groups according to BMI of 25 kg/m^2^ and cutoff value of TyG index of 8.8. In addition, for comparison we also divided subjects by BMI of 25 kg/m^2^ and the modified criteria for metabolic health proposed by Wildman et al.[5, 26]

As shown in Table 3 and S2 Table, when subjects were divided by TyG index, the number of subjects with TyG < 8.8 was higher in those who were metabolically unhealthy (MUHO or MUHNO) than in those who were metabolically healthy (MHNO or MHO). In subjects with BMI < 25 kg/m^2^, it was 6.2% higher (1,445 vs. 1,361 subjects), compared with a 22.9% increase in subjects with BMI ≥ 25 kg/m^2^ (424 vs. 345 subjects).

If subjects were divided by the TyG cutoff value of 8.8, patients with TyG <8.8 had more metabolically unhealthy parameters in both the obese and non-obese subgroups. There was a similar pattern of trends of metabolic parameters in four groups divided by the two methods of TyG and metabolic health. Few differences were found between the groups of MUHNO and MHO. Cox analysis showed a significantly increased risk for future diabetes in all groups except for the MHO group and the group with BMI ≥ 25 kg/m^2^ and TyG < 8.8 (Table 4). Therefore, the TyG index seems to be an independent parameter for assessment of metabolic abnormality, comparable to the accepted definition of metabolic health.

In addition to insulin resistance, abnormalities in body composition and body fat distribution, i.e., an increase in total fat mass, body fat percentage, subcutaneous fat, and visceral fat, were found in MUHNO individuals in previous studies. In addition, decreased fat storage in adipose tissue was associated with increased fat storage in non-physiological depots such as liver and muscle[31–33]. Given that body composition measurements were carried out by segmental bioelectric impedance, we could not examine visceral fat deposition. In this study, a mild increase in body fat mass in subjects with TyG index ≥ 8.8 was seen, but there was a mild decrease in percent body fat. Similar findings were found in our subjects with metabolically unhealthy status (Table 3 and S2 Table). These findings could be explained by the fact that decreased fat storage in adipose tissue and increased fat storage in non-physiological depots such as liver and muscle results in increased plasma triglyceride concentrations. Although we did not examine visceral fat distribution, we predict that the subjects with TyG index ≥ 8.8 had increased visceral fat distribution.

TG or fasting glucose levels, used for calculation of TyG index, could be a predictor of development of diabetes[34–36]. However, Navarro-Gonzalez et al. showed that the predicting value of TyG index was more superior than that of TG or fasting glucose in individuals with normal fasting glucose, with the higher HRs and the areas under the ROC curves value[37]. So TyG index is more suitable for screening than TG or fasting glucose.

Our study had several strengths. First, all subjects were observed for 5 years. Second, as our subjects were asymptomatic young and middle-aged males and females our study may be less likely to be affected by selection bias, reverse causation, and confounding factors of comorbidities and medication use than studies based on older populations[38]. Second, we used carefully standardized high-quality clinical and laboratory procedures. Third, this study involves detailed clinical and biochemical assessment, allowing adjustment for potential confounders. We also considered hsCRP, representing subclinical inflammation.

In addition to these strengths, however, several limitations of this study should be considered. First, the lack of 2-h postload glucose test is a limitation because it might have resulted in inclusion of subjects with undiagnosed diabetes at baseline. Second, we did not consider the use of other drugs for dyslipidemia in our analysis, which might have influenced glucose levels. Third, our study was based on Korean adults therefore further studies of other races are needed for generalization of our findings.

In conclusion, in this retrospective longitudinal study we aimed to investigate whether the TyG index could be a valuable marker for predicting diabetes and to compare it with other methods used to define metabolic health. The TyG index measured at a single time point may be an indicator of the risk for incident diabetes. Moreover, the predictive value of the TyG index was comparable to that of metabolic health.

## Supporting Information

S1 Fig Receiver operating characteristic curves for the TyG index predicting incident diabetes. (TIF)

S1 Table Baseline characteristics of total subjects. (DOCX)

S2 Table Baseline characteristics and their comparisons according to obesity status and metabolic health. (DOCX)

## References

[1] Yusuf S, Hawken S, Ounpuu S, Bautista L, Franzosi MG, Commerford P, et al Obesity and the risk of myocardial infarction in 27,000 participants from 52 countries: a case-control study. Lancet. 2005;366(9497):1640–9. 10.1016/S0140-6736(05)67663-5 . 16271645

[2] Hubert HB, Feinleib M, McNamara PM, Castelli WP. Obesity as an independent risk factor for cardiovascular disease: a 26-year follow-up of participants in the Framingham Heart Study. Circulation. 1983;67(5):968–77. Epub 1983/05/01. . 6219830 10.1161/01.cir.67.5.968

[3] Mokdad AH, Ford ES, Bowman BA, Dietz WH, Vinicor F, Bales VS, et al Prevalence of obesity, diabetes, and obesity-related health risk factors, 2001. Jama. 2003;289(1):76–9. Epub 2002/12/31. . 12503980 10.1001/jama.289.1.76

[4] Rhee EJ, Lee MK, Kim JD, Jeon WS, Bae JC, Park SE, et al Metabolic health is a more important determinant for diabetes development than simple obesity: a 4-year retrospective longitudinal study. PLoS One. 2014;9(5):e98369 Epub 2014/05/30. 10.1371/journal.pone.0098369 24870949 PMC4037196

[5] Wildman RP, Muntner P, Reynolds K, McGinn AP, Rajpathak S, Wylie-Rosett J, et al The obese without cardiometabolic risk factor clustering and the normal weight with cardiometabolic risk factor clustering: prevalence and correlates of 2 phenotypes among the US population (NHANES 1999–2004). Arch Intern Med. 2008;168(15):1617–24. Epub 2008/08/13. 10.1001/archinte.168.15.1617 . 18695075

[6] Appleton SL, Seaborn CJ, Visvanathan R, Hill CL, Gill TK, Taylor AW, et al Diabetes and cardiovascular disease outcomes in the metabolically healthy obese phenotype: a cohort study. Diabetes Care. 2013;36(8):2388–94. Epub 2013/03/16. 10.2337/dc12-1971 23491523 PMC3714523

[7] Lee SH, Yang HK, Ha HS, Lee JH, Kwon HS, Park YM, et al Changes in Metabolic Health Status Over Time and Risk of Developing Type 2 Diabetes: A Prospective Cohort Study. Medicine (Baltimore). 2015;94(40):e1705 10.1097/MD.0000000000001705 26448024 PMC4616763

[8] Kuk JL, Ardern CI. Are metabolically normal but obese individuals at lower risk for all-cause mortality? Diabetes Care. 2009;32(12):2297–9. 10.2337/dc09-0574 19729521 PMC2782994

[9] Aung K, Lorenzo C, Hinojosa MA, Haffner SM. Risk of developing diabetes and cardiovascular disease in metabolically unhealthy normal-weight and metabolically healthy obese individuals. J Clin Endocrinol Metab. 2014;99(2):462–8. 10.1210/jc.2013-2832 24257907 PMC3913817

[10] Choi KM, Cho HJ, Choi HY, Yang SJ, Yoo HJ, Seo JA, et al Higher mortality in metabolically obese normal-weight people than in metabolically healthy obese subjects in elderly Koreans. Clin Endocrinol (Oxf). 2013;79(3):364–70. 10.1111/cen.12154 . 23330616

[11] Arnlov J, Ingelsson E, Sundstrom J, Lind L. Impact of body mass index and the metabolic syndrome on the risk of cardiovascular disease and death in middle-aged men. Circulation. 2010;121(2):230–6. 10.1161/CIRCULATIONAHA.109.887521 . 20038741

[12] Seo MH, Rhee EJ. Metabolic and cardiovascular implications of a metabolically healthy obesity phenotype. Endocrinology and metabolism (Seoul, Korea). 2014;29(4):427–34. Epub 2015/01/07. 10.3803/EnM.2014.29.4.427 ; PubMed Central PMCID: PMCPmc4285032. 25559571 PMC4285032

[13] Lee MK, Rhee EJ, Kim MC, Moon BS, Lee JI, Song YS, et al Metabolic Health Is More Important than Obesity in the Development of Nonalcoholic Fatty Liver Disease: A 4-Year Retrospective Study. Endocrinology and metabolism (Seoul, Korea). 2015;30(4):522–30. Epub 2015/09/24. 10.3803/EnM.2015.30.4.522 ; PubMed Central PMCID: PMCPmc4722408. 26394730 PMC4722408

[14] Hamer M, Stamatakis E. Metabolically healthy obesity and risk of all-cause and cardiovascular disease mortality. J Clin Endocrinol Metab. 2012;97(7):2482–8. Epub 2012/04/18. 10.1210/jc.2011-3475 22508708 PMC3387408

[15] Lee SH, Han K, Yang HK, Kim HS, Cho JH, Kwon HS, et al A novel criterion for identifying metabolically obese but normal weight individuals using the product of triglycerides and glucose. Nutr Diabetes. 2015;5:e149 10.1038/nutd.2014.46 25915739 PMC4423196

[16] Simental-Mendia LE, Rodriguez-Moran M, Guerrero-Romero F. The product of fasting glucose and triglycerides as surrogate for identifying insulin resistance in apparently healthy subjects. Metab Syndr Relat Disord. 2008;6(4):299–304. 10.1089/met.2008.0034 . 19067533

[17] Lee SH, Kwon HS, Park YM, Ha HS, Jeong SH, Yang HK, et al Predicting the development of diabetes using the product of triglycerides and glucose: the Chungju Metabolic Disease Cohort (CMC) study. PLoS One. 2014;9(2):e90430 10.1371/journal.pone.0090430 24587359 PMC3938726

[18] Guerrero-Romero F, Simental-Mendia LE, Gonzalez-Ortiz M, Martinez-Abundis E, Ramos-Zavala MG, Hernandez-Gonzalez SO, et al The product of triglycerides and glucose, a simple measure of insulin sensitivity. Comparison with the euglycemic-hyperinsulinemic clamp. J Clin Endocrinol Metab. 2010;95(7):3347–51. 10.1210/jc.2010-0288 . 20484475

[19] Janssen I, Heymsfield SB, Ross R. Low relative skeletal muscle mass (sarcopenia) in older persons is associated with functional impairment and physical disability. J Am Geriatr Soc. 2002;50(5):889–96. . 12028177 10.1046/j.1532-5415.2002.50216.x

[20] Methods. LoNC. Available at http://www.ngsp.org/docs/methods.pdf. Lastly accessed 23rd July, 2016.

[21] Schwartz KL, Monsur JC, Bartoces MG, West PA, Neale AV. Correlation of same-visit HbA1c test with laboratory-based measurements: a MetroNet study. BMC Fam Pract. 2005;6:28 10.1186/1471-2296-6-28 16014170 PMC1185531

[22] Matthews DR, Hosker JP, Rudenski AS, Naylor BA, Treacher DF, Turner RC. Homeostasis model assessment: insulin resistance and beta-cell function from fasting plasma glucose and insulin concentrations in man. Diabetologia. 1985;28(7):412–9. . 3899825 10.1007/BF00280883

[23] Chobanian AV, Bakris GL, Black HR, Cushman WC, Green LA, Izzo JL Jr., et al Seventh report of the Joint National Committee on Prevention, Detection, Evaluation, and Treatment of High Blood Pressure. Hypertension. 2003;42(6):1206–52. 10.1161/01.HYP.0000107251.49515.c2 . 14656957

[24] American Diabetes A. Standards of medical care in diabetes—2014. Diabetes Care. 2014;37 Suppl 1:S14–80. 10.2337/dc14-S014 . 24357209

[25] Steering Committee of the WHO Western Pacific Region II. The Asia–Pacific perspective: redefining obesity and its treatment, Australia, 2000.

[26] Alberti KG, Eckel RH, Grundy SM, Zimmet PZ, Cleeman JI, Donato KA, et al Harmonizing the metabolic syndrome: a joint interim statement of the International Diabetes Federation Task Force on Epidemiology and Prevention; National Heart, Lung, and Blood Institute; American Heart Association; World Heart Federation; International Atherosclerosis Society; and International Association for the Study of Obesity. Circulation. 2009;120(16):1640–5. 10.1161/CIRCULATIONAHA.109.192644 . 19805654

[27] Naf S, Escote X, Ballesteros M, Yanez RE, Simon-Muela I, Gil P, et al Serum activin A and follistatin levels in gestational diabetes and the association of the Activin A-Follistatin system with anthropometric parameters in offspring. PLoS One. 2014;9(4):e92175 10.1371/journal.pone.0092175 24763182 PMC3998926

[28] Tripathy D, Almgren P, Tuomi T, Groop L. Contribution of insulin-stimulated glucose uptake and basal hepatic insulin sensitivity to surrogate measures of insulin sensitivity. Diabetes Care. 2004;27(9):2204–10. Epub 2004/08/31. . 15333485 10.2337/diacare.27.9.2204

[29] Kelley DE, Goodpaster BH, Storlien L. Muscle triglyceride and insulin resistance. Annu Rev Nutr. 2002;22:325–46. 10.1146/annurev.nutr.22.010402.102912 . 12055349

[30] Ruderman N, Chisholm D, Pi-Sunyer X, Schneider S. The metabolically obese, normal-weight individual revisited. Diabetes. 1998;47(5):699–713. Epub 1998/05/20. . 9588440 10.2337/diabetes.47.5.699

[31] Karelis AD, St-Pierre DH, Conus F, Rabasa-Lhoret R, Poehlman ET. Metabolic and body composition factors in subgroups of obesity: what do we know? J Clin Endocrinol Metab. 2004;89(6):2569–75. Epub 2004/06/08. 10.1210/jc.2004-0165 . 15181025

[32] Conus F, Allison DB, Rabasa-Lhoret R, St-Onge M, St-Pierre DH, Tremblay-Lebeau A. Metabolic and behavioral characteristics of metabolically obese but normal-weight women. J Clin Endocrinol Metab. 2004;89:5013–20. 15472199 10.1210/jc.2004-0265

[33] Katsuki A, Sumida Y, Urakawa H, Gabazza EC, Murashima S, Maruyama N. Increased visceral fat and serum levels of triglyceride are associated with insulin resistance in Japanese metabolically obese, normal weight subjects with normal glucose tolerance. Diabetes Care. 2003;26:2341–4. 12882859 10.2337/diacare.26.8.2341

[34] Miller M, Stone NJ, Ballantyne C, Bittner V, Criqui MH, Ginsberg HN, et al Triglycerides and cardiovascular disease: a scientific statement from the American Heart Association. Circulation. 2011;123(20):2292–333. Epub 2011/04/20. 10.1161/CIR.0b013e3182160726 . 21502576

[35] Tirosh A, Shai I, Tekes-Manova D, Israeli E, Pereg D, Shochat T, et al Normal fasting plasma glucose levels and type 2 diabetes in young men. N Engl J Med. 2005;353(14):1454–62. Epub 2005/10/07. 10.1056/NEJMoa050080 . 16207847

[36] Nichols GA, Hillier TA, Brown JB. Normal fasting plasma glucose and risk of type 2 diabetes diagnosis. Am J Med. 2008;121(6):519–24. Epub 2008/05/27. 10.1016/j.amjmed.2008.02.026 . 18501234

[37] Navarro-Gonzalez D, Sanchez-Inigo L, Pastrana-Delgado J, Fernandez-Montero A, Martinez JA. Triglyceride-glucose index (TyG index) in comparison with fasting plasma glucose improved diabetes prediction in patients with normal fasting glucose: The Vascular-Metabolic CUN cohort. Preventive medicine. 2016;86:99–105. Epub 2016/02/09. 10.1016/j.ypmed.2016.01.022 . 26854766

[38] Chang Y, Ryu S, Choi Y, Zhang Y, Cho J, Kwon MJ, et al Metabolically Healthy Obesity and Development of Chronic Kidney Disease: A Cohort Study. Ann Intern Med. 2016;164(5):305–12. 10.7326/M15-1323 . 26857595

