# Supplementary figures and images for: Predictive Value of Triglyceride Glucose Index for the Risk of Incident Diabetes: A 4-Year Retrospective Longitudinal Study
Source: PLoS One. 2016 Sep 28;11(9):e0163465. doi: 10.1371/journal.pone.0163465 (PMC5040250; doi:10.1371/journal.pone.0163465)

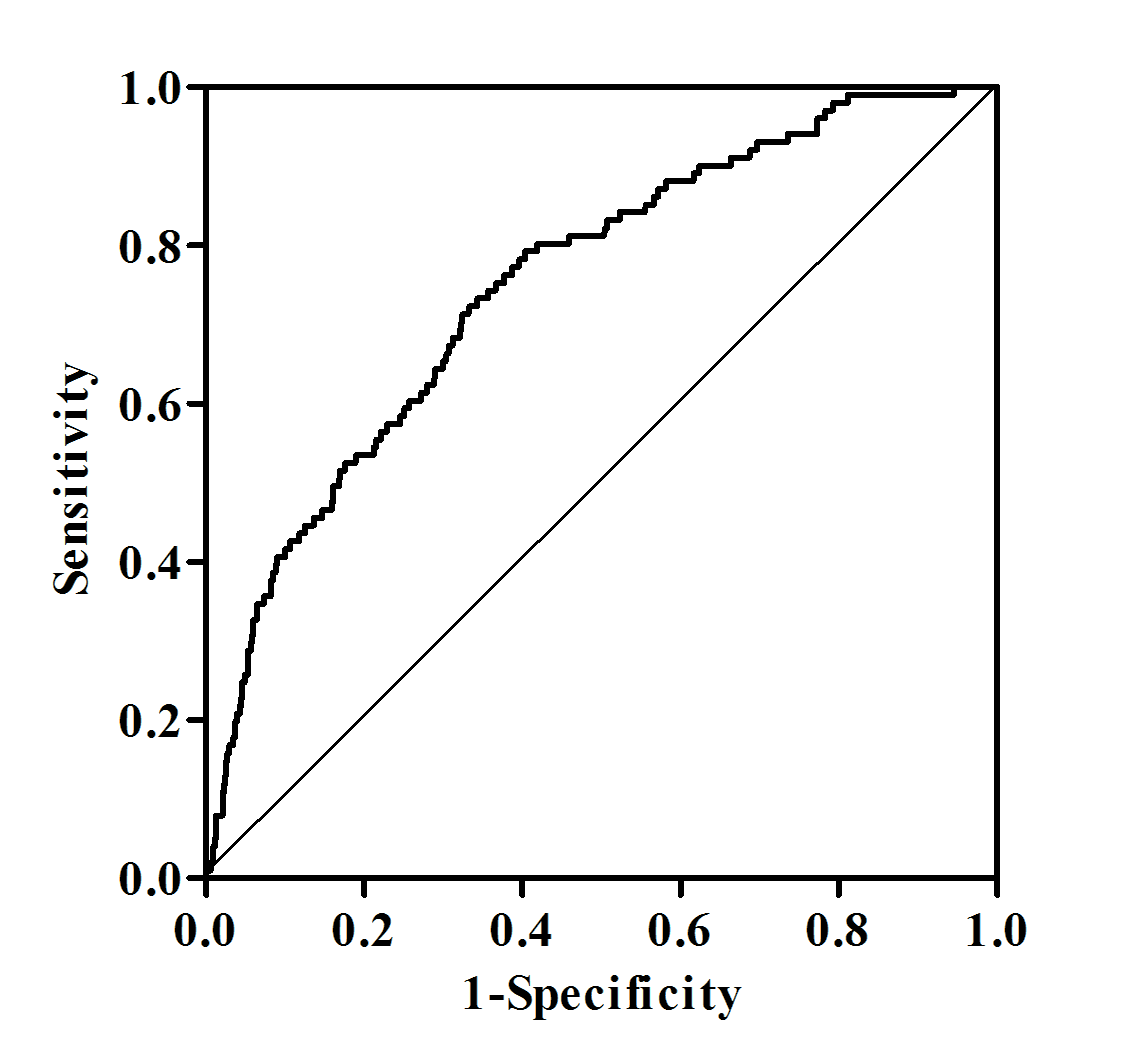

Supplement: S1 Fig — (TIF) [file pone.0163465.s001.tif]
