# Supplementary material for: Predictive Value of Triglyceride Glucose Index for the Risk of Incident Diabetes: A 4-Year Retrospective Longitudinal Study
Source: PLoS One. 2016 Sep 28;11(9):e0163465. doi: 10.1371/journal.pone.0163465 (PMC5040250; doi:10.1371/journal.pone.0163465)
Supplement: S1 Table — (DOCX) [file pone.0163465.s002.docx]

S1 Table. Baseline characteristics of total subjects

| Variables (N=2,900) | Value |
| --- | --- |
| Age (years) | 44.3 ± 6.5 |
| Sex, male (%) | 2078 (71.7) |
| BMI (kg/m^2^) | 23.8 ± 2.9 |
| < 18.5 kg/m^2^ | 68 (2.3) |
| 18.5 ~ 25 kg/m^2^ | 1888 (65.1) |
| ≥ 25 kg/m^2^ | 944 (32.6) |
| Waist circumference (cm) | 80.9 ± 9.0 |
| Lean mass (kg) | 48.2 ± 8.5 |
| Body fat mass (kg) | 16.3 ± 4.6 |
| Percent body fat (%) | 24.3 ± 5.5 |
| Systolic BP (mmHg) | 112.7 ± 14.6 |
| Diastolic BP (mmHg) | 76.5 ± 10.3 |
| Total cholesterol (mg/dL) | 194.5 ± 33.3 |
| Triglyceride (mg/dL) | 133.1 ± 84.0 |
| HDL-C (mg/dL) | 53.0 ± 12.0 |
| LDL-C (mg/dL) | 112.2 ± 27.6 |
| HbA1c (%) | 5.4 ± 0.3 |
| Fasting glucose (mg/dl) | 95.7 ± 8.7 |
| Fasting insulin (IU/L) | 8.8 ± 3.4 |
| HOMA-IR | 2.10 ± 0.89 |
| hsCRP (mg/dL) | 0.12 ± 0.41 |
| Smoking (%)^a^ | 1517 (53.3) |
| Alcohol drinking (%) | 307 (10.6) |
| Regular exercise (%) | 647 (22.3) |
| IFG (%) | 836 (28.8) |
| Metabolically unhealthy status (%) | 1194 (41.2) |
| TyG index | 8.61 ± 0.54 |

Data are presented as frequency (%), or mean ± standard deviation.
BMI, body mass index; BP, blood pressure; AST, aspartate aminotransferase; ALT, alanine aminotransferase; BUN, blood urea nitrogen; HDL-C, high-density lipoprotein cholesterol; LDL-C, low-density lipoprotein cholesterol; HbA1c, glycosylated hemoglobin; HOMA-IR, homeostasis model assessment index - insulin resistance; hsCRP, high-sensitivity C-reactive protein; IFG, impaired fasting glucose
^a^ Subjects who have ever smoked more than 5 packs of cigarettes.
 Smoking history was available only in 2845 subjects.
