# Supplementary material for: Predictive Value of Triglyceride Glucose Index for the Risk of Incident Diabetes: A 4-Year Retrospective Longitudinal Study
Source: PLoS One. 2016 Sep 28;11(9):e0163465. doi: 10.1371/journal.pone.0163465 (PMC5040250; doi:10.1371/journal.pone.0163465)
Supplement: S2 Table — (DOCX) [file pone.0163465.s003.docx]

**S2 Table. Baseline characteristics and their comparisons according to obesity status and metabolic health**

| Variable | MHNO  (n=1361) | MUHNO  (n=595) | MHO  (n=345) | MUHO  (n=599) | *P value^*^* |
| --- | --- | --- | --- | --- | --- |
| Age (years) | 43.9 ± 6.6 | 45.0 ± 6.7^§^ | 44.4 ± 6.0 | 44.5 ± 6.2 | 0.009 |
| Sex, male (%) | 751 (55.2) | 488 (82.0)^§^ | 279 (80.9)^#^ | 560 (93.5)^#,§^ | < 0.001 |
| BMI (kg/m^2^) | 21.8 ± 1.9 | 23.1 ± 1.5^§^ | 26.5 ± 1.3^#^ | 27.2 ± 1.8^#,§^ | < 0.001 |
| Waist circumference (cm) | 75.2 ± 7.4 | 80.8 ± 6.1^§^ | 87.7 ± 5.9^#^ | 89.9 ± 5.6^#,§^ | < 0.001 |
| Lean mass (kg) | 44.1 ± 7.7 | 48.4 ± 7.0^§^ | 52.4 ± 7.5^#^ | 55.1 ± 6.1^#,§^ | < 0.001 |
| Body fat mass (kg) | 13.9 ± 3.4 | 15.1 ± 2.9^§^ | 20.1 ± 3.8^#^ | 20.9 ± 3.9^#,§^ | < 0.001 |
| Percent body fat (%) | 23.2 ± 5.8 | 22.9 ± 4.8 | 26.9 ± 5.5^#^ | 26.5 ± 4.2^#^ | < 0.001 |
| Systolic BP (mmHg) | 107.2 ± 12.3 | 117.0 ± 15.1^§^ | 111.8 ± 11.5^#^ | 121.3 ± 15.0^#,§^ | < 0.001 |
| Diastolic BP (mmHg) | 72.0 ± 8.8 | 79.3 ± 10.0^§^ | 76.9 ± 8.2^#^ | 83.5 ± 10.0^#,§^ | < 0.001 |
| Total cholesterol (mg/dL) | 189.1 ± 30.9 | 195.8 ± 34.9^§^ | 200.4 ± 31.4^#^ | 201.9 ± 35.8^#^ | < 0.001 |
| Triglyceride (mg/dL) | 92.5 ± 37.3 | 174.8 ± 99.8^§^ | 117.5 ± 47.8^#^ | 192.9 ± 103.3^#,§^ | < 0.001 |
| HDL-C (mg/dL) | 58.0 ± 11.8 | 47.6 ± 10.3^§^ | 54.0 ± 10.1^#^ | 46.3 ± 8.9^#,§^ | < 0.001 |
| LDL-C (mg/dL) | 108.1 ± 25.8 | 111.5 ± 28.8^§^ | 119.8 ± 26.6^#^ | 117.7 ± 29.2^#^ | < 0.001 |
| HbA1c (%) | 5.4 ± 0.3 | 5.4 ± 0.4^§^ | 5.4 ± 0.3 | 5.5 ± 0.3^#,§^ | < 0.001 |
| Fasting glucose (mg/dl) | 92.3 ± 7.2 | 99.2 ± 8.8^§^ | 93.8 ± 6.6^#^ | 101.0 ± 8.9^#,§^ | < 0.001 |
| Fasting insulin (IU/L) | 7.5 ± 2.3 | 9.0 ± 3.3^§^ | 8.9 ± 2.7^#^ | 11.4 ± 4.1^#,§^ | < 0.001 |
| HOMA-IR | 1.72 ± 0.56 | 2.22 ± 0.83^§^ | 2.05 ± 0.65^#^ | 2.86 ± 1.12^#,§^ | < 0.001 |
| hsCRP (mg/dL) | 0.10 ± 0.39 | 0.11 ± 0.27^§^ | 0.14 ± 0.40^#^ | 0.18 ± 0.54^#,§^ | < 0.001 |
| Smoking (%)^a^ | 520 (39.2) | 367 (63.0)^§^ | 199 (58.4)^#^ | 431 (72.7)^#,§^ | < 0.001 |
| Alcohol drinking (%) | 108 (7.9) | 78 (13.1)^§^ | 44 (12.8)^#^ | 77 (12.9)^#^ | < 0.001 |
| Regular exercise (%) | 322 (23.7) | 123 (20.7) | 78 (22.6) | 124 (20.7) | 0.353 |
| IFG (%) | 157 (11.5) | 313 (52.6)^§^ | 37 (10.7) | 329 (54.9)^#,§^ | < 0.001 |
| TyG index | 8.29 ± 0.39 | 8.93 ± 0.49^#,§^ | 8.54 ± 0.37^#^ | 9.07 ± 0.47^#,§^ | < 0.001 |

Data are presented as frequency (%), mean ± standard deviation.
BMI, body mass index; MHNO, metabolically healthy non-obese; MUHNO, metabolically unhealthy non-obese; MHO, metabolically healthy obese; MUHO, metabolically unhealthy obese; BP, blood pressure; AST, aspartate aminotransferase; ALT, alanine aminotransferase; BUN, blood urea nitrogen; HDL-C, high-density lipoprotein cholesterol; LDL-C, low-density lipoprotein cholesterol; *HbA1c*, glycosylated hemoglobin; HOMA-IR, homeostasis model assessment index - insulin resistance; hsCRP, high-sensitivity C-reactive protein; IFG, impaired fasting glucose
^a^ Subjects who have ever smoked more than 5 packs of cigarettes.
 Smoking history was available only in 2845 subjects, divided into groups of 1328, 583, 341, and 593 subjects.
^§^ *p* < 0.05, in comparison with the group with TyG < 8.8 from one-way ANOVA analysis.
^#^ *p* < 0.05, in comparison with the reference group (MHNO) from one-way ANOVA analysis.
*P* values were corrected by Bonferroni’s method due to multiple testing.
AST, ALT, triglyceride, fasting insulin, and hsCRP were converted to Ln values and HOMA-IR was converted to square root value for the analysis.
